# *AGER* expression and alternative splicing in bronchial biopsies of smokers and never smokers

**DOI:** 10.1186/s12931-019-1038-6

**Published:** 2019-04-10

**Authors:** Alen Faiz, Maarten van den Berge, Cornelis J. Vermeulen, Nick H. T. ten Hacken, Victor Guryev, Simon D. Pouwels

**Affiliations:** 1 Department of Pathology & Medical Biology, University Medical Center Groningen, University of Groningen, Hanzeplein 1, 9713 GZ Groningen, The Netherlands; 2 Groningen Research Institute for Asthma and COPD, University Medical Center Groningen, Univesity of Groningen, Hanzeplein 1, 9713 GZ Groningen, The Netherlands; 3 Department of Pulmonary Diseases, University Medical Center Groningen, University of Groningen, Groningen, The Netherlands; 4 European Research Institute for the Biology of Ageing, University of Groningen, Groningen, The Netherlands

**Keywords:** Cigarette smoking, COPD, RAGE, AGER, Alternative splicing

## Abstract

Cigarette smoking is one of the major risk factors for the development of chronic obstructive pulmonary disease (COPD). Evidence is accumulating that Receptor for Advanced Glycation-End products (RAGE)-signaling is a key pathway in the pathophysiology of COPD. To date, it is unknown how smoking affects RAGE expression. In the current study, we investigated the effect of smoking on *AGER*, the gene encoding RAGE, expression and on alternative splicing of *AGER*. To this end, we conducted RNA-Seq on bronchial biopsies for asymptomatic smokers (*n* = 36) and never smokers (*n* = 40). Total *AGER* gene expression was accessed using DESeq2, while alternative splicing was investigated by measuring the number of specific split reads spanning exon-exon junctions and the total split reads. One of the major isoforms of RAGE is endogenous soluble (es) RAGE, an anti-inflammatory decoy receptor, making up for approximately 10% of the total amount of soluble (s)RAGE. We found that smokers show decreased total gene expression of *AGER* in bronchial biopsies, while the relative abundance of the esRAGE isoform is increased. Furthermore, no difference in the serum levels of total sRAGE were observed between smokers and non-smokers. Our data indicates that smoking initiates a protective anti-inflammatory mechanism with decreased expression of the pro-inflammatory gene *AGER* and increased relative abundance of the anti-inflammatory isoform esRAGE.

## To the editor

To date, cigarette smoking is one of the biggest public health threats worldwide, being causative for the dead of approximately 7 million people every year, according to the World Health Organization (WHO). Smoking can lead to multiple diseases, including cardiovascular diseases, lung cancer and chronic obstructive pulmonary disease (COPD). The underlying mechanisms of these diseases are still largely unknown. However, there seems to be one molecular pathway which connects the three diseases, the receptor for advanced glycation end-products (RAGE) signaling pathway. RAGE is a pro-inflammatory pattern recognition receptor, which is mainly expressed by type I alveolar cells. The freely circulating soluble form of RAGE (sRAGE), inhibits RAGE signaling by binding RAGE ligands and preventing homo-dimerization of RAGE, necessary for activation. sRAGE can be formed by proteolytic cleavage or by alternative splicing. In the latter case the transmembrane domain, encoded by exon 10 of the gene encoding RAGE, *AGER*, is spliced out, resulting in the endogenous soluble form of RAGE (esRAGE), which passes the cell membrane and is released into the extracellular space upon translation.

In cardiovascular diseases it has been shown that RAGE signaling triggers and maintains the inflammatory state, and sRAGE can be used as a biomarker for the development of cardiovascular diseases [1], while in lung cancer RAGE expression may be beneficial, acting as a tumor-suppressor [2], However, the role of RAGE signaling is best characterized in COPD. The gene encoding RAGE, has been shown to be a susceptibility gene for COPD [3]. Furthermore, sRAGE levels are decreased in COPD patients [4], while the levels of several RAGE ligands are increased both locally and systemically [5], indicating increased RAGE signaling. However, the effect of smoking on RAGE signaling is less well studied. It was shown that stimulation of gingival carcinoma epithelial cells with cigarette smoke extract (CSE) increases RAGE expression and the subsequent inflammatory reaction and induces oral squamous cell carcinoma cell invasion [6]. Furthermore, it was shown that RAGE knock-out mice had reduced cigarette smoke-induced neutrophilic airway inflammation [7]. However, there is less consensus about the effect of smoking on the circulating sRAGE levels, with some studies reporting no significant differences in serum sRAGE levels between smokers and never smokers [8], while other studies show a decreased level of sRAGE in serum of smokers [9]. One study even showed an increase in the serum sRAGE levels of smokers compared to never smokers [10]. In the current study we investigated the gene expression levels of *AGER* in lung tissue of smokers and never smokers and subsequently evaluated the level of alternative splicing in these individuals. Alternative splicing was measured using a novel, in house developed technique, based on comparing the number of specific split reads spanning exon-exon junctions to the total split reads per gene.

**Fig. 1:**
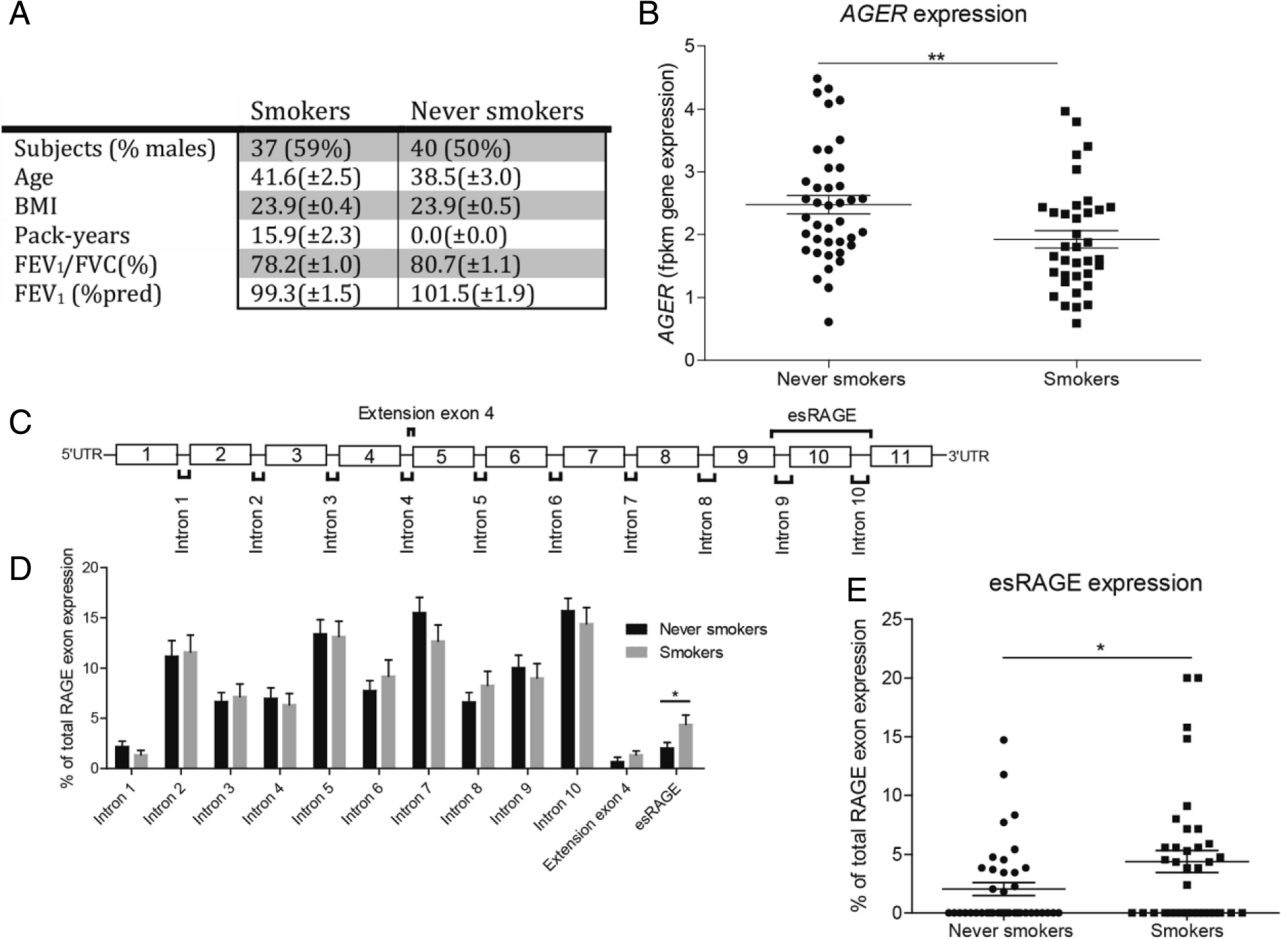
Smokers show lower AGER expression and increased esRAGE expression. **a** Patient characteristics of 37 active smokers and 40 never smokers used for all experiments in this study. BMI = body mass index as kg/m2, Packyears = number of years smoking one pack of 20 cigarettes per day, FVC = Forced vital capacity, FEV_1_ = Forced expiratory volume in one second. Data is shown as mean ± SEM. **b** AGER mRNA expression was measured in lung tissue obtained from bronchial biopsy material in smokers and never smokers without airway obstruction. Gene expression expressed as Fragments Per Kilobase Million (fpmk). **c** Graphical representation of the AGER exon structure and sequences spanning the exon-exon junctions (split reads). **d** Percentage of expression of split reads compared to total split read count per patient for all exon-exon junctions and alternative splicing sites. **e** Percentage of expression of split reads compared to total split read count per patient for esRAGE. All data is shown as individual measurements and mean ± SEM. Significance was tested using a Mann Whitney-U test,* *p* ≤ 0.05, ** *p* < 0.01

Here, we used bronchial biopsies from 37 active smokers and 40 never smokers without airway obstruction, which were matched based on age, sex, body mass index and lung function (ClinicalTrials.gov Identifier: NCT00848406) [11]. Clinical variables are shown in Fig. 1a. All study protocols were approved by the medical ethic committee of the University Medical Center Groningen (UMCG), Groningen, The Netherlands and all subjects provided written informed consent. Furthermore, all clinical procedures were performed according to the standards set by the latest Declaration of Helsinki. Bronchial biopsies were taken from segmental divisions of the main bronchi. Total RNA was extracted from the biopsies, converted to cDNA and the obtained cDNA fragment libraries were loaded in pools of multiple samples in an Illumina HiSeq2500 sequencer using default parameters for paired-end sequencing (2 × 100 bp). Quality control (QC) metrics were calculated for the raw sequencing data, using the FastQC tool (version 0.11.3). The trimmed FASTQ files were aligned to human reference genome GRCh37 using HISAT (version 0.1.5) allowing for 2 mismatches. Before gene quantification SAMtools (version 1.2) was used to sort the aligned reads. The gene level quantification was performed by HTSeq (version 0.6.1p1) using Ensembl version 75 as gene annotation database. Gene expression analysis was conducted using raw counts of *AGER* and analyzed using the R-package DESeq2. Feature counts were set as the dependent variable, smoking status was investigated correcting for age and gender. The splice site usage was quantified by counting split reads mapping across exon-exon junctions using a custom perl script (available upon request). We processed BAM alignment files and skipped reads marked as PCR duplicates. For each intron in the alignment (flag N in CIGAR alignment string) we recorded the chromosome and first and last intron base. Afterwards, we quantified the number of reads for all observed intron positions across alignment files from all samples.

Here, we showed a significant decrease of total *AGER* expression in smokers compared to never smokers (Fig. 1b, smokers 1.92 ± 0.03 fpkm, never smokers 2.48 ± 0.02 fpkm, Mann Whitney-U test, *p* = 0.006). To investigate whether smoking affects the expression of endogenous soluble RAGE (esRAGE), all splicing events within the *AGER* gene were measured. We were able to detect all annotated intron splicing sites, an extension of exon 4 and the splicing of exon 10 which leads to the production of esRAGE (Fig. 1c). For all normal intron splicing sites and the alternative splice variant leading to an extension of exon 4, we did not observe any differences between smokers and never smokers (Fig. 1d). Interestingly, the splicing of exon 10 leading to the production of esRAGE was found to be increased within smokers (Fig. 1d-e*, smokers 3.55 ± 0.12%, never smokers 2.44 ± 0.09%, Mann Whitney-U test, P = 0.03*). Thus, the total expression of *AGER* is decreased in bronchial tissue of smokers, whereas the fraction of RAGE which leads to esRAGE is increased.

While studies on the effect of smoking on RAGE expression are limited, in vitro and murine studies have shown that exposure of lung epithelial cells to cigarette smoke extract or mice to cigarette smoke increased the expression of RAGE [12]. Moreover, in a study investigating the RAGE expression in never smokers and active smokers using immunohistochemistry, an increase in RAGE expression was shown in mucosal cells specifically [12]. Previously, up to 19 different alternative splice variants of *AGER* were detected on mRNA level in in vitro cultured human cells [12]. In the current study, we employed a novel technique to detect alternative splice variants of *AGER* in human bronchial biopsies. Here, we were able to detect two alternative splice variants of RAGE, an elongation of the fourth exon and the splicing out of exon 10. Our study is the first to show differences in alternative splicing of *AGER* in smokers and never smokers. Here, it was shown that the expression of the pro-inflammatory RAGE receptor decreases and the relative portion of *AGER* expression which leads to the production of the anti-inflammatory decoy receptor esRAGE, increases. This suggests an anti-inflammatory mechanism to protect against an exaggerated immune reaction upon smoke exposure. Dysregulation of these anti-inflammatory mechanisms may trigger smoke-related diseases, including cardiovascular diseases, lung cancer and COPD. However, in the current population we also found that CD8+ T-cells were increased in current smokers compared to never-smokers (*data not shown*), as low levels of *AGER* expression have been observed in CD8+ T-cells before, we cannot exclude the possibility of enhanced splicing of the transcript that leads to esRAGE in CD8+ T-cells, influencing our results.

In summary, this is the first study investigating the expression of all splice sites within the *AGER* gene in relation to the total *AGER* gene expression. Here, we showed that although smokers have lowered total *AGER* expression, the portion of *AGER*, which leads to the production of the anti-inflammatory esRAGE protein is increased. Our study provides new insight in the molecular mechanisms of the human body to protect against the harmful effects of cigarette smoking. Upon dysregulation of these processes the susceptibility for smoke-induced diseases may increase and restoring the balance in the RAGE pathway may be utilized as potential therapeutic target in several smoke-related pathologies.
